# Sex-differential reproduction success and selection on floral traits in gynodioecious *Salvia pratensis*

**DOI:** 10.1186/s12870-019-1972-y

**Published:** 2019-08-27

**Authors:** Bo Zhang, Regine Claßen-Bockhoff

**Affiliations:** 1 Key Laboratory of Grassland Ecosystem of Ministry of Education, and Sino-U.S. Centers for Grazingland Ecosystem Sustainability, College of Grassland Science, Gansu Agricultural University, Lanzhou, 730070 China; 2 Institute of Organismic and Molecular Evolution, Johannes Gutenberg-University, 55099 Mainz, Germany

**Keywords:** Evolutionary divergence, Female advantage, Gynodioecy, Pollination, Phenotypic selection, Reproductive performance, *Salvia*, Sexual dimorphism

## Abstract

**Background:**

Gynodioecy, a sexual system with hermaphrodite and female individuals in a population, raises the question how the two sexual morphs are maintained. *Salvia pratensis* is a gynodioecious species featured by its modified stamens that act as a lever mechanism in pollination. Given sexual dimorphism in floral traits of the species, it is predictable that two sexual morphs differ in their interplay with pollinators and thus in their fitness. In this study, we investigated sex-specific reproduction success and floral adaptation in a population of *S. pratensis*.

**Results:**

We found that two sexual morphs in *S. pratensis* distinctly differed in their floral proportions. Female flowers fitted better to the pollinators than hermaphrodites in terms of touching the stigmas when being probed, and hence were more efficient in pollen deposition. Floral traits overall underwent stronger selection in the population, with stigma position and corolla length subject to disruptive selection mediated by different body-sized bumble bees; some selections on floral traits were significantly different in the strength, even opposite in the direction between two morphs. Flower production tended to be under correlational selection with floral structural traits, implying that a large plant with many flowers did not show an advantage in fitness unless its flower construction mechanically matched the pollinators well.

**Conclusions:**

In conclusion, the pollinator-mediated selection likely played an important role in the evolution and maintenance of sexual dimorphism in the gynodioecious *S. pratensis*; and sex-divergent mechanical interaction with pollinators served as a critical mechanism by which female individuals were maintained in the population with a female advantage in pollen deposition efficiency (i.e. receiving pollen).

## Background

Gynodioecy is a dimorphic sexual system, in which hermaphroditic and female individuals coexist within a population [1]. It is rare, but widely distributed in angiosperm [2]. It occurs in at least 81 angiosperm families, but in far less than 1% of angiosperm species [2–4]. Hermaphrodites can transmit their genes via both seeds and pollen, whereas females do it only via seeds [1]. Because of this fitness disadvantage of females, it has been an intriguing topic in evolutionary biology to understand the evolution of gynodioecy, particularly the mechanism of maintaining females in gynodioecious population. Theoretically, it is believed that a female advantage, i.e. a higher female fitness in females over hermaphrodites, is necessary to compensate for the loss of male function in female individuals [5].

Female advantage can be, first, achieved by higher seed set due to more resources which are otherwise reallocated to pollen production [6]. Second, the female can gain the advantage of fitness by avoiding inbreeding depression [7, 8], which is sometimes considered as the main process responsible for gynodioecy [9]. Theoretically, female advantage can also occur by sex-differential interactions with biotic factors (reviewed by [5]), such as sex-biased seed predation [10, 11] and nectar robbing [12]. However, it has not been reported that the sex-specific interplay with pollinators can contribute to female advantage in fitness. On the contrary, the disadvantages in females have been documented much more frequently, which are mainly caused by low pollinator attraction [13–15] and/or lack of pollen as a reward [16–19].

Pollinator-mediated selection has been widely believed to play a key role in floral divergence not only among populations (or species) [20], but also within a population [21, 22]. Gender dimorphism, i.e. between-sex floral divergence beyond sexual organs, universally exists in gynodioecious species [18, 23]. Considering close interactions between flowers and pollinators in the process of pollination, it is predictable that both sexual morphs in gynodioecious population differ in their interplay with pollinators, and thereby subject to different selection pressure on floral traits. Hermaphrodite flowers are usually larger and more attractive than the females due to their greater allocation to attraction (e.g. petal size, nectar or pollen) [24, 25]. Thus, it is likely that the hermaphrodite evolves toward functional male provided it benefits more in male function than female function from increased pollinator visitation [26, 27]. Apart from sexual difference in pollination attraction, the sex-differential mechanical fitting to pollinators could be another source that generates difference in reproductive success between sexes. Therefore, it will be informative to investigate sex-specific adaption of floral traits by pollination ecology and phenotypic selection, and can provide insights into the evolutionary processes and maintenance of sexual dimorphism in gynodioecy.

**Fig. 1:**
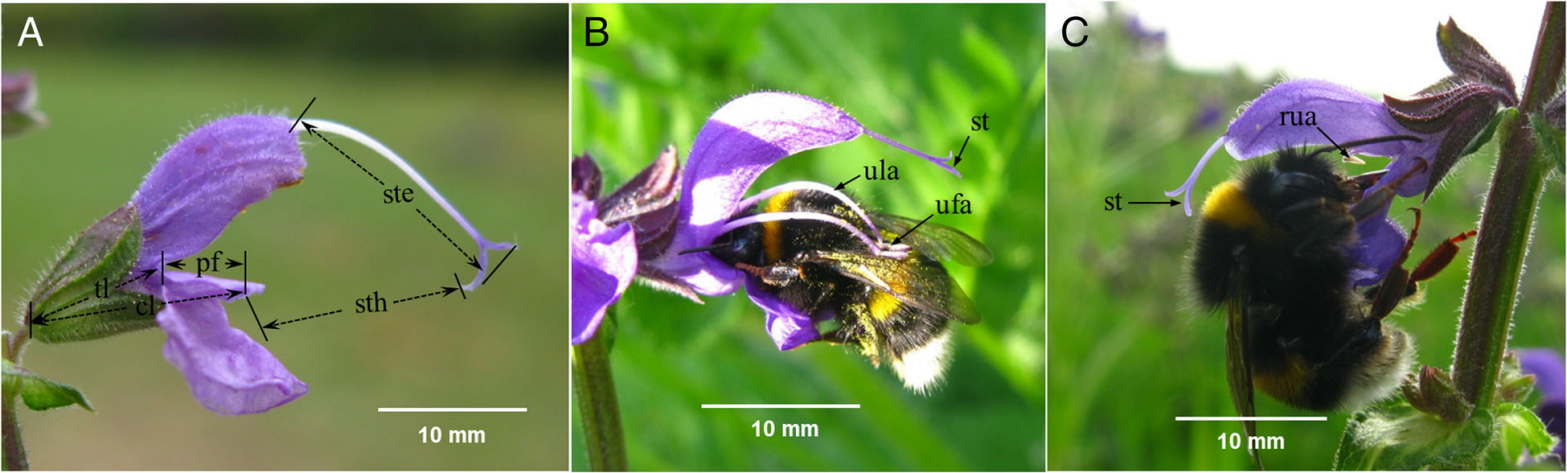
Floral traits and dorsal pollination in *Salvia pratensis*. Flower morphometrics (**a**), dorsal pollinations for hermaphrodites (**b**) and females (**c**). cl, corolla length; tl, corolla tube length; ste, style exsertion; sth, style height; st, stigma; ufa, upper fertile anther; ula, upper lever arm; rua, reduced upper anther

*Salvia pratensis* is a perennial gynodioecious species mainly distributed in Europe. The species is self-compatible with a mixed mating system [28], and primarily pollinated by bumble bees and/or honey bees [29, 30], (Fig. 1). As with most species in the genus, two stamens of each hermaphrodite flower are modified to lever-like structures functioning as a lever mechanism in pollination, with the lower theca of each stamen reduced and the upper one fertile [31], (Fig. 1b). In female flowers, the thecae or even entire upper arms of the stamens are reduced, resulting in a nonfunctional lever mechanism. Regarding the lever mechanism, it has been argued that the interplay between flowers and pollinators is vulnerable against variation in each of the interactive parts, and that floral traits are under strong selection, as already documented in *S. digitaloides* [32], (Fig. 1c). This means, minute changes in the proportions of the pollinators or floral structures may have significant consequences for pollination success and hence the fitness [29].

In the present study, we presume that the gynodioecious *S. pratensis* may serve as an ideal model for the study on the evolution of sexual dimorphism. Given that floral traits are different between sexes, we predict that two sexual morphs exhibit different interactions with pollinators, and thus different pollination efficiency and reproduction success. Consequently, the floral traits will be subject to sex-differential selection mediated by the pollinators. Specifically, we address the following three questions. 1) Do floral traits exhibit sexual dimorphism in gynodioecious *S. pratensis*? 2) How does selection act on floral traits and differ between sexual morphs? 3) What is the mechanism of maintaining females in the gynodioecious population?

## Results

### Sexual dimorphism and pollinator assemblage in the gynodioecious *S. pratensis*

Floral traits significantly differed between two sexual morphs in the population of *S. pratensis* (Table 1). The female had on average a shorter corolla and tube, a smaller platform (i.e. flower mouth), and a stigma closer to the platform than hermaphrodite flowers. Style exsertion, flower production and stalk diameter did not significantly differ between two morphs.

**Table 1:** Morphometric data of floral traits in gynodioecious *Salvia pratensis*

Floral traits (mm)	Gynodioecious population		Comparison between sexual morphs *P* values
	Hermaphrodites (n = 42)	Females (n = 36)	
Corolla	**20.44 ± 0.28**	**16.85 ± 0.22**	**< 0.0001**
Tube	**9.15 ± 0.12**	**7.98 ± 0.09**	**< 0.0001**
Platform	**11.30 ± 0.19**	**8.87 ± 0.15**	**< 0.0001**
Style exsertion	6.58 ± 0.25	6.23 ± 0.18	0.278
Style height	**11.27 ± 0.21**	**9.63 ± 0.20**	**< 0.0001**
Flower no.	49.79 ± 1.27	48.5 ± 1.79	0.551
Stalk diameter	2.43 ± 0.06	2.30 ± 0.05	0.093

Mean values (± SE) of floral traits are in mm except for the flower number per inflorescence. Trait values in bold are significantly different between hermaphrodite and female flowers with ANOVA

**Fig. 2:**
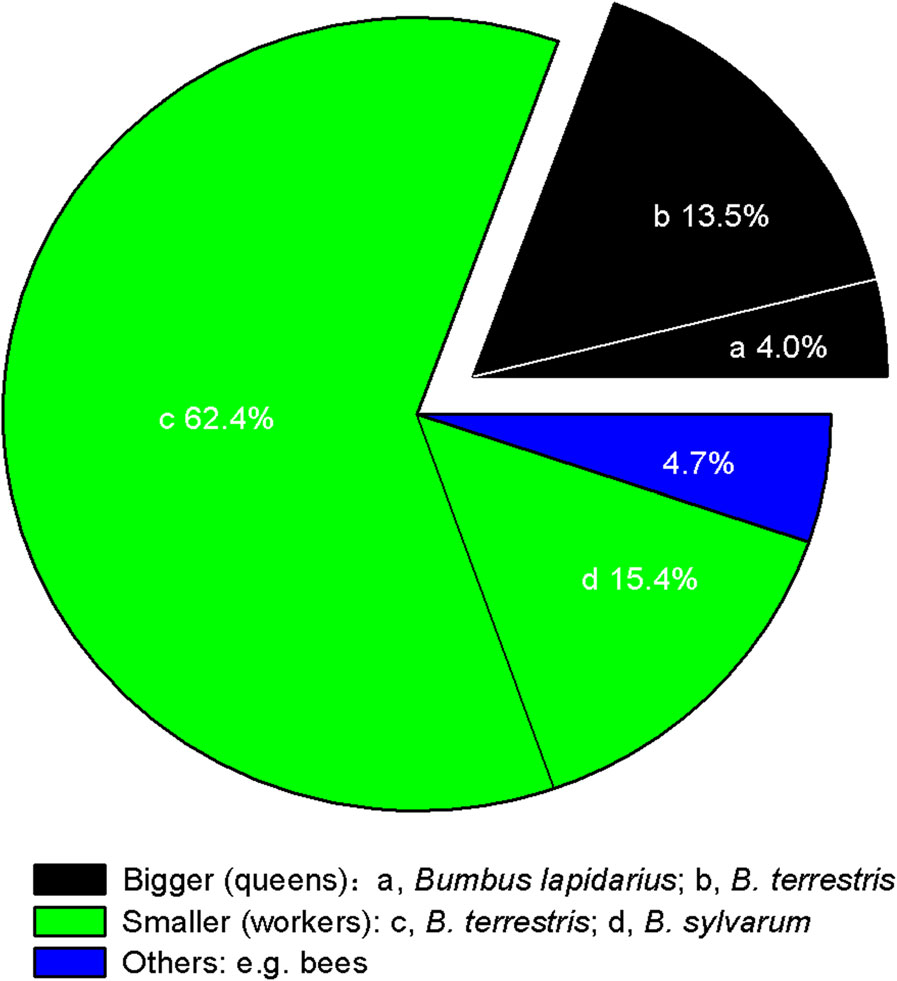
Pollinator assemblage in gynodioecious *Salvia pratensis*

Pollinator assemblage in the population consisted of both queens and workers of *B. terrestris*, workers of *B. sylvarum* and queens of *B. lapidarius*. Among them, *B. terrestris* was the dominant species, with a frequency of more than 75.9% (Fig. 2). Workers of *B. terrestris* had a mean body length of 16.07 ± 0.63 mm, thorax thickness of 5.38 ± 0.22 mm and tongue length of 4.14 ± 0.21 mm (n = 4). The queens of *B. terrestris* and *B. lapidaries* accounted for 17.4% of all bumblebees. They were distinctly bigger than the other pollinators, and the body length, thorax thickness and tongue length of *B. terrestris* queens were 27.2 mm, 7.82 mm and 5.15 mm, respectively.

### Sex-differential pollination and reproduction success in *S. pratensis*

**Fig. 3:**
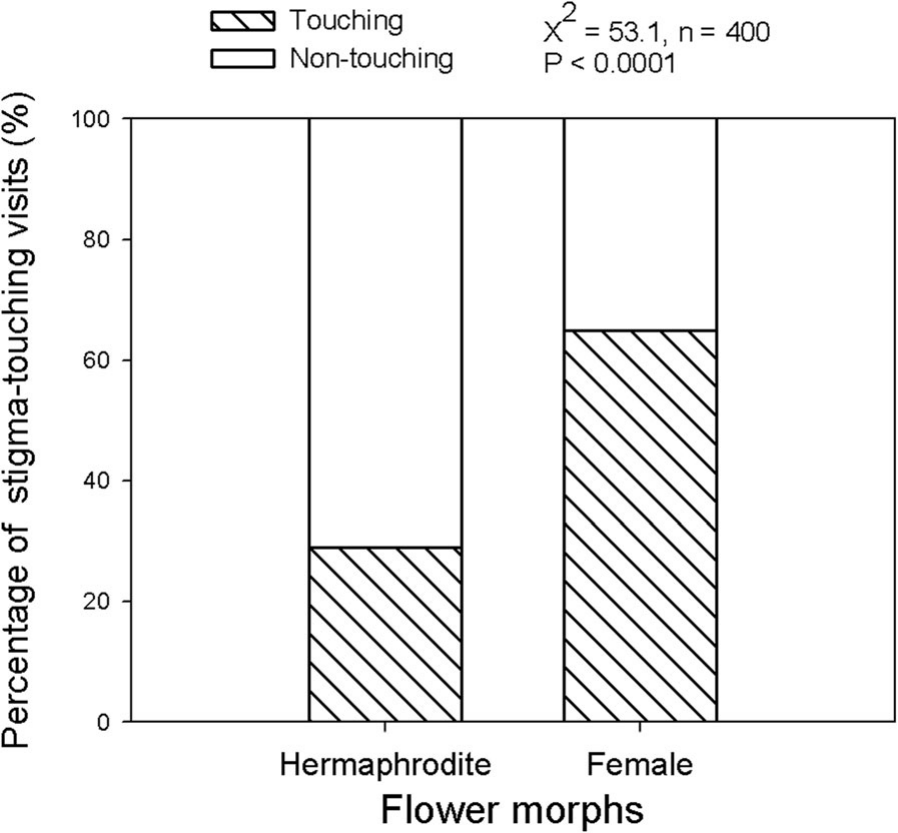
Difference in pollination efficiency (by *Bombus terrestris*) between two sexual morphs in gynodioecious *Salvia pratensis*

The queens of bumble bees were distinctly more successful in touching stigma when foraging than the workers given their relatively bigger body proportions. The successful visits, i.e. the probes touching the stigmas, accounted for 86.3% of total probes (n = 87). Two sexual morphs significantly differed in the percentage of successful visits by the dominant pollinators (i.e. *B. terrestris*) (Fig. 3). For the female flowers, the percentage of touching-stigma visits was over 65% in total visits, whereas the value was less than 30% for the hermaphrodite flowers.

**Fig. 4:**
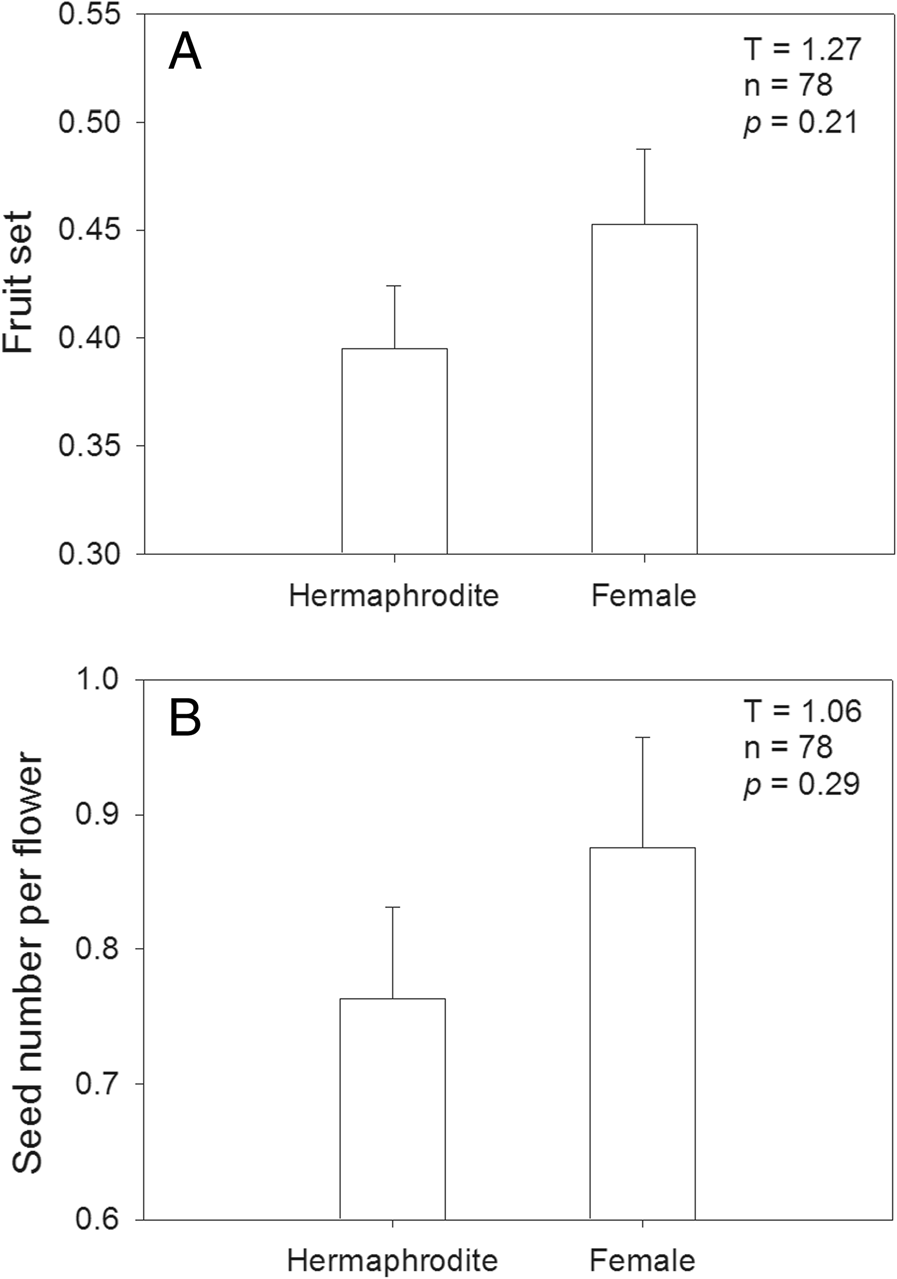
Fruit set (**a**) and seed (nutlet) set per flower (**b**) of two sexual morphs in gynodioecious *Salvia pratensis*

As showed in Fig. 4, the hermaphrodite individuals in the population had a fruit set of 39.5% ± 2.9% (mean ± se, n = 42), and seed set per flower of 0.76 ± 0.07 (mean ± se, n = 42). Female individuals had a higher fruit set of 45.3% ± 3.5% (mean ± se, n = 36), and higher seed set of 0.87 ± 0.08 (mean ± se, n = 36) than the hermaphrodites; but each of them did not significantly differ between two morphs.

### Sex-differential selection on floral traits in *S. pratensis*

The complete model for selection gradient analysis explained about 86.1% of total variation in female fitness among hermaphrodite group (F = 5.88, *p* = 0.0001), and 85.4% of total variation among female group. The ANCOVA model for selection comparison between two morphs was also highly significant, explaining about 87.8% of total variation (F = 2.88, *p* = 0.004).

**Fig. 5:**
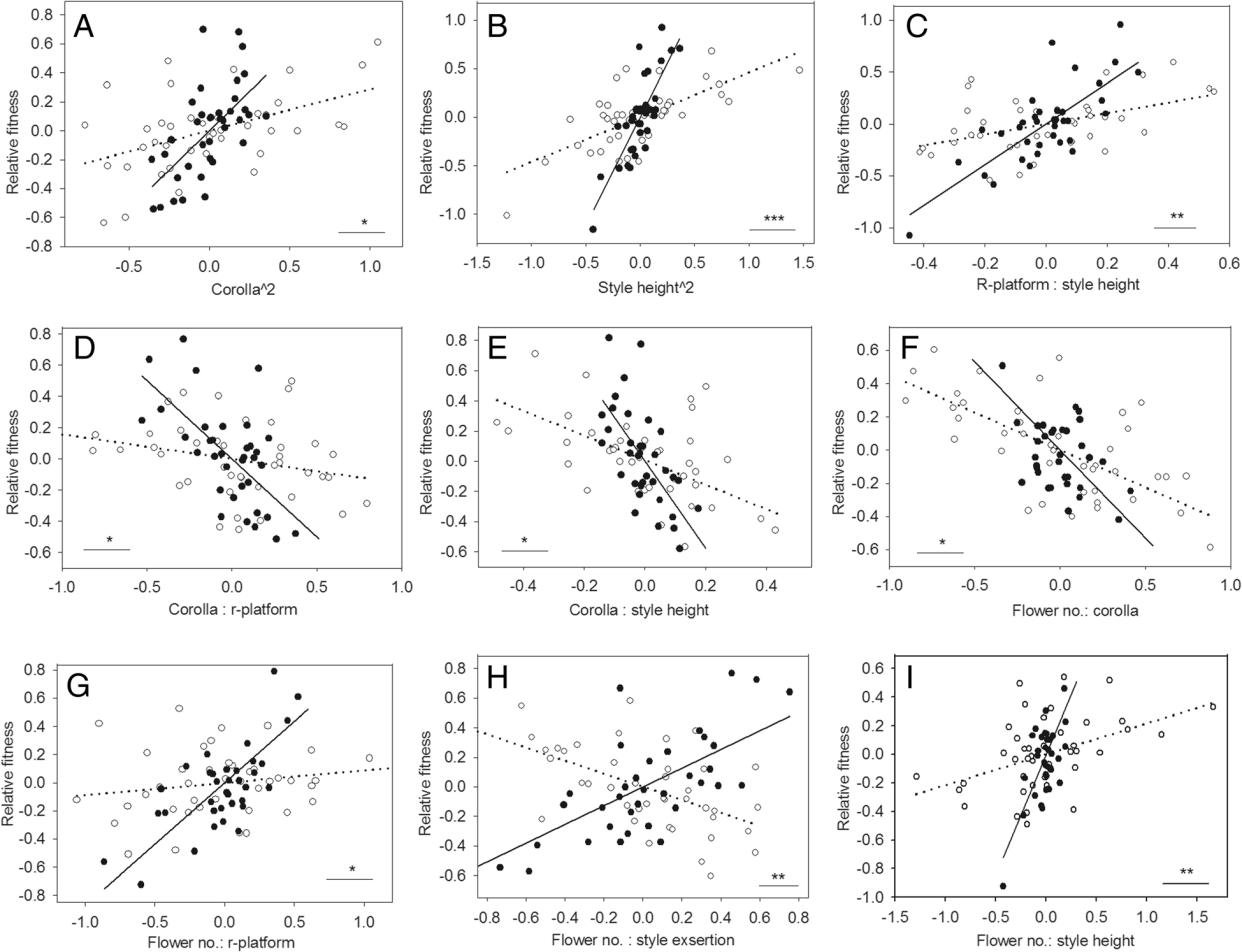
Standardized phenotypic selection gradients that were significantly different between two sexual morphs in gynodioecious Salvia pratensis (**a**-**i**). Selection gradients were illustrated with the added-variable plots, in which the residuals from a complete model of relative fitness on all traits except the focal trait are plotted against the residuals from a regression model of the focal trait on the other traits. The relationships between relative fitness and trait values were illustrated for hermaphrodite morph with open symbols and dashed line, and for female morph with filled symbols and solid line. The asterisks indicate significant difference between morphs: “*” for *p* < 0.05, “**” *p* < 0.01, and “***” *p* < 0.001

Among five floral traits determined, only style exsertion was subject to significant directional selection among female plants in the population. Its gradient was 0.52 ± 0.20 (*p* = 0.034), but not significantly different with that among hermaphrodite plants. As for nonlinear selection, positive quadratic selection was consistently detected on corolla length among each sexual group. The selection gradient was 2.17 ± 0.96 (*p* = 0.05) among female individuals, significantly higher than 0.58 ± 0.23 (*p* = 0.022) among hermaphrodites (Table 2, Fig. 5a). Significant quadratic selection was also detected on style exsertion among hermaphrodite individuals (*p* = 0.006), but the selection gradient did not differ significantly with the female’s. Strong quadratic selection acted consistently on style height in both sexual groups. The selection gradient for the female was 4.43 ± 1.13 (*p* = 0.004), significantly higher than 0.92 ± 0.22 (*p* < 0.001) for the hermaphrodite (Table 2, Fig. 5b).

**Table 2:** Selection gradients for floral traits and comparison between sexual morphs in *Salvia pratensis*

Explanatory variables included in the complete model ^a^	Hermaphrodite		Female		Comparison between morphs ^c^	
	Selection gradients (±se)	*P* value	Selection gradients (±se)	*P* value	Interaction coefficient (±se)	*P* value
Directional selection						
Corolla	−0.10 ± 0.11	0.387	0.68 ± 0.35	0.091	−2.42 ± 1.32	0.08
R-platform	− 0.18 ± 0.10	0.09	− 0.23 ± 0.30	0.452	−0.36 ± 0.52	0.494
Style exsertion	0.00 ± 0.11	0.991	**0.52 ± 0.20**	**0.034**	−0.67 ± 0.53	0.219
Style height	0.17 ± 0.11	0.137	−0.88 ± 0.43	0.075	1.21 ± 1.14	0.299
Flower number	−0.01 ± 0.11	0.952	− 0.21 ± 0.22	0.365	− 0.07 ± 0.41	0.857
Nonlinear selection ^b^						
Corolla ^ 2	**0.58 ± 0.23**	**0.022**	**2.17 ± 0.96**	**0.053**	−3.09 ± 1.31	**0.027**
R-platform ^ 2	−0.04 ± 0.09	0.691	0.39 ± 0.39	0.355	− 0.36 ± 0.27	0.199
Style exsertion ^ 2	**0.63 ± 0.2**	**0.006**	0.22 ± 0.29	0.46	0.12 ± 0.25	0.65
Style height ^ 2	**0.92 ± 0.22**	**0.0004**	**4.43 ± 1.13**	**0.004**	−3.23 ± 0.76	**0.0003**
Flower no. ^ 2	−0.16 ± 0.15	0.292	0 ± 0.61	0.998	− 0.03 ± 0.27	0.922
Correlational selection						
Corolla × r-platform	−0.16 ± 0.12	0.194	**− 1.00 ± 0.39**	**0.033**	2.19 ± 0.78	**0.01**
Corolla × style exsertion	**0.77 ± 0.21**	**0.001**	0.18 ± 0.39	0.667	0.69 ± 0.83	0.415
Corolla × style height	**−0.92 ± 0.24**	**0.001**	**−2.88 ± 1.05**	**0.025**	5.55 ± 2.01	**0.011**
Corolla × flower no	**− 0.45 ± 0.11**	**0.0006**	**−1.06 ± 0.44**	**0.042**	1.51 ± 0.72	**0.047**
R-platform × style exsertion	**− 0.46 ± 0.13**	**0.0029**	−0.44 ± 0.37	0.271	0.36 ± 0.50	0.479
R-platform × style height	**0.52 ± 0.22**	**0.0289**	**1.96 ± 0.58**	**0.01**	−2.73 ± 0.85	**0.004**
R-platform × flower no	0.09 ± 0.10	0.3839	**0.87 ± 0.29**	**0.017**	−0.82 ± 0.35	**0.03**
Style exsertion × style height	−0.16 ± 0.16	0.339	**− 0.74 ± 0.32**	**0.049**	0.89 ± 0.48	0.077
Style exsertion × flower no.	**− 0.44 ± 0.14**	**0.004**	**0.63 ± 0.23**	**0.026**	− 1.05 ± 0.33	**0.004**
Style height × flower no.	**0.22 ± 0.10 (N)**	**0.04**	**1.69 ± 0.68**	**0.038**	− 1.85 ± 0.64	**0.009**

^a^ The terms related to stalk diameter in the model were not presented. ^b^ Each value of nonlinear selection gradients was the double of coefficient for each squared term. ^c^ Difference in selection gradients between two sexual morphs was examined with ANCOVA, indicated by significant coefficient of interaction between each term and flower type. All values in bold indicated the significance of statistics. “N” in brackets after selection gradient indicated that no distinct selection was reflected by the added-variable plot, although the selection gradient was significant statistically.

Positive correlational selection was detected on style height and platform, and negative correlational selection on style height and corolla length among either sex-morph individuals; the selection gradient for each combination significantly differed between two sexual morphs (Table 2, Fig. 5c, e). Style exsertion and corolla length was subject to positive correlational selection only among the hermaphrodites, with a selection gradient of 0.77 ± 0.21 (*p* = 0.001), which was not significantly different from that among the females. Negative correlational selection was detected on the combination of style exsertion and platform among hermaphrodite individuals. Marginally significant correlational selection was detected on the combination of style height and exsertion among female group. Negative correlational selection was consistently detected on flower number (i.e. flower production) and corolla length among either sex-morph group. The selection gradient was − 0.45 ± 0.22 (*p* = 0.0006) among the hermaphrodites, significantly higher than − 1.06 ± 0.44 (*p* = 0.042) among the females (Table 2, Fig. 5f). Positive correlational selection was consistently detected on flower production and style height among either sexual group; however, the added-variable plot reflected distinct selection only among female individuals, and there existed significant difference between two morphs (Table 2, Fig. 5i). The combination of flower production and platform was favored only among female group, subject to positive correlational selection. Flower production and style exsertion underwent opposite correlational selection among different sex-morph group (Fig. 5h). The selection gradient was − 0.44 ± 0.14 among the hermaphrodites, whereas it was 0.63 ± 0.23 among the females (Table 2).

## Discussion

### Evolutionary divergence in floral traits of gynodioecious *S. pratensis*

Gynodioecious species usually exhibit gender divergence in their floral traits. Often, the size of flowers is larger in hermaphrodites than in females [18, 23]. In present study, it was also found that floral traits (e.g. corolla, tube length and stigma height) were significantly larger in hermaphrodites than in females of *S. pratensis*. There have been two nonexclusive explanations for the evolution of dimorphism in flower size of gynodioecious species. Firstly, the species with few ovules usually have relatively small female flowers [18], because they either rarely suffer from pollen limitation and thus need not to invest too much in flower size for pollinator attraction, or have less floral structures to protect [24, 33]. Secondly, small female flowers likely contribute to female advantage by saving resources for seed production (i.e. resource compensation) [34, 35], while the larger hermaphroditic flowers enhance pollinator attraction and thereby male function [36, 37].

Apart from floral size, flower production was also usually sexually dimorphic to different extent in dimorphic species [38, 39]. However, no evidence indicated sexual dimorphism of flower production in the *S. pratensis* population. Besides the influence of sampling on similarity of plant size in the study, highly intersexual genetic correlation could be one of the main reasons for the absence of sexual dimorphism in flower number [40], which constrained its divergence between two sexual morphs. Further, it was also reasonable that two sexual morphs had relatively consistent flower production in the gynodioecious population. Because flower number of an inflorescence usually affects floral display and thus pollinator attraction, the similar production of flowers in both morphs may assure pollinators foraging all individuals in a sex-unbiased way [19]. Otherwise, if either sexual morph has a much smaller floral display than the other, the resulting decreased visitation by pollinators will affect pollen export for the hermaphrodites, i.e. male fitness, and reduce the opportunity and efficiency of pollination for the females.

### Sex-specific reproduction success in gynodioecious *S. pratensis*

Theoretically, female advantage is a prerequisite for the maintenance of females in a gynodioecious population to compensate for the lost male function [1, 2]. In present study, the female flowers had relatively smaller floral structures (particularly, a lower stigma position) such that they matched distinctly better with the pollinators than did the hermaphrodite flowers. In consequence, the female individuals had an advantage over the hermaphrodites in pollen deposition onto stigmas, i.e. receiving pollen for the realization of female fitness. However, both fruit and seed sets in the females were only slightly higher than those in the hermaphrodites. The reason was probably that the female was prone to pollen limitation due to the lack of reproductive assurance through self-pollination [19, 41]. In the population studied, each sexual morph of *S. pratensis* had a large floral display, and bumble bees tended to probe flowers in sequence within an inflorescence (also see [42]). Therefore, it was likely that the females underwent local pollen depletion (i.e. carryover) [43] when pollinators constantly probed flowers within or among female plants [44, 45]. As such, the female advantage in pollen deposition might be counteracted by pollen limitation, provided no adequate pollen import.

Although theoretically required for the gynodioecy, female advantage was not always found in natural populations of gynodioecious species [5], such as *Beta vulgaris* [46], *Daphne laureola* [47] and *Raphanus sativus* [48]. For the absence of female advantage, a convincing explanation is that these populations are being in a phase of evolutionary dynamics, which is featured by a high frequency of females. As discussed above, the females are prone to suffer from pollen limitation in such case [49] and hence have a declined and even undetectable female advantage due to overall drop in pollen availability [5]. Nevertheless, such a slight female advantage observed in the *S. pratensis* population does not still violate the least requirement for the maintenance of nuclear-cytoplasmic gynodioecy [50, 51], although no detailed information has been known on the sex determination of the species.

### Selection on floral traits in gynodioecious *S. pratensis*

Sex-specific selection has been considered as one of the primary evolutionary processes responsible for the evolution and maintenance of sexual dimorphism, interplaying with the breakdown of intersexual genetic correlations that constrain between-sex divergence of traits [40, 52]. In present study, although the sample size was relatively small, significant selections were still detected on floral traits in the gynodioecious *S. pratensis*; and the strength and pattern of selections for some traits exhibited distinct differences between both sexual morphs.

Flower production determines the capacity of plants for mating opportunities through male and female functions, and thus, tends to be under positive directional selection [53, 54]. However, in present study, significantly directional selection was not detected on flower production among either sex-morph individuals. That is, female fitness did not positively respond to the increase in flower production. Partially, the sampling of plants with similar size in the study might have artificially reduced variation in flower number in both sexual groups, and therefore potentially hided evidence for selection on flower number. Besides, severe pollen limitation might be another cause, due to high frequency of females in the gynodioecious population. In such case, the positive correlation between female fitness and flower production would become weak and even disappeared (see [42]). This argument was further supported by the findings of correlational selections on flower number and other floral traits as followed.

In the population, flower production (i.e. flower number) tended to be under correlational selection with floral structural traits, like platform, and style height and exsertion determining stigma position. This implied that the contribution of flower production to female fitness was affected by a given construction of flowers in the plant. Among hermaphrodite individuals, negative correlational selection acted on flower number and style exsertion, indicating that a large plant with many flowers would has a high female fitness given with a relatively short style exsertion. As hermaphrodite flowers were significantly larger in floral structures than female flowers, such as longer corolla and larger platform (i.e. relatively larger flower mouth), thereby, bumble bees could land and enter the flowers in a horizontal manner when probing. In this case, a relatively shorter style exsertion could facilitate bumble bees probing the flowers, having the stigma positioned right above bumble bee’s back and not at flower entrance as a barrier. In contrast, female flowers were distinctly small in structures, particularly in platform; thereby, bumble bees landed the flowers in a vertical manner and hung on the low lip when probing. In this case, it was not surprising that selection favored positive correlations between flower number with platform size, style exsertion or stigma high among female plants, as the findings showed. That is, a large platform and/or relatively far stigma from platform were beneficial to the fitness realization of a large female plant. It was reasonable because such a flower construction could not only provide enough space for bumble bee landing by a horizontal way, but also make the stigma easier to touch. Taken together, given a large inflorescence, many more flowers produced could not be transformed into the advantage in female fitness unless the flower construction mechanically fitted pollinators well.

Finally, our findings indicated that stigma height tended to be under disruptive selection among either hermaphrodite or female individuals, and the selection strength was much stronger in the female group. It was implied that the plants with either high- or low-positioned stigma were equally favored in the population. Meanwhile, flower size (i.e. corolla length) was also subject to disruptive selection among either sex-morph individuals. Considering two types of bumble bees with different body size in the population, it could be reasonably interpreted that the two types of flowers stood equal chance of pollination success due to their divergent adaption to both sizes of bumble bees. Besides, significantly correlational selection for platform and style height also reflected divergent adaptation of the trait pair to different pollinators. The flowers with a large platform and a high stigma could fit well to big bumble bees, whereas the flowers with the two smaller traits fit better to small bumble bees. As such, the disruptive selection mediated by pollinators likely played an important role in the evolution and maintenance of sexual dimorphism in the gynodioecious *S. pratensis*.

## Conclusions

Gynodioecy has long drawn biologist’s attention to the question how females are maintained in the population. Theoretically, a female advantage is necessary for the maintenance of gynodioecy. In the study, we found that floral traits significantly differed between two sexual morphs in the gynodioecious *S. pratensis*. Sexual divergence in flower size conferred female individuals an advantage in pollen deposition (i.e. receiving pollen) over the hermaphrodites, because relatively smaller flowers of the female’s fit better to the pollinators. However, the females just gained a slightly higher fitness than the hermaphrodites due to their intrinsic disadvantage in pollen availability during pollination; that is, pollen limitation could be one of the main reasons for the weak difference in female fitness between two sexual morphs. Therefore, it was predictable that the female advantage in fitness varied with population dynamics (e.g. sex ratio) and pollinator’s activity. Floral traits overall underwent strong selection in the gynodioecious population, with flower size and stigma position subject to disruptive selection. Flower production tended to be under correlational selection with floral structural traits, implying that many more flowers in a large plant could not be transformed into the advantage in fitness unless the flower construction mechanically matched pollinators well (i.e. efficient in pollination). In conclusion, the pollinator-mediated selection likely played an important role in the evolution and maintenance of sexual dimorphism in the gynodioecious *S. pratensis*, and the sex-divergent mechanical interaction with pollinator served as a mechanism by which female individuals, with an advantage in pollen deposition efficiency, were maintained in the gynodioecious population.

## Materials and methods

### Study species and site

The study was conducted during the main flowering season of *Salvia pratensis* in May 2016 at Mainz, Germany. The studied population was located close to a small shrubbery in the midst of farmland at Mainz-Hechtsheim (49°56′23″N, 8°15′04″E; 197 m a.s.l.), with an area of 45 × 3.5 m^2^. The whole area was densely covered by individuals of *S. pratensis* (over 500 individuals). The female and hermaphrodite individuals were almost evenly distributed in the population, and their relative proportion (i.e. sex ratio) was close to 50%.

### Experimental approach

In the mid of the flowering season, we randomly labeled 80 individuals (44 hermaphrodites and 36 females) in the gynodioecious population. These labeled plants were about the same size and flowered synchronously. For morphometric measurements, three or four completely opened flowers were randomly selected in each labeled inflorescence as the representatives of the plant. Five floral structural traits were measured in each flower (see Fig. 1): corolla length (cl), corolla tube length (tl), style height (sth) defined as the distance between the lower lip and the stigma, style exsertion (se), and platform size determined by subtracting “cl” from “tl”. The diameter of the main axis under the first branch was measured as a proxy of plant size.

Each tagged plant was collected about 20 days after flower withering when nutlets were matured but still enclosed in the calyx. Flower number was determined by counting the number of pedicels which remained on the inflorescence after flower withering. Seed (nutlet) number per inflorescence was counted as an estimate of female fitness component for each plant. All traits were measured by using digital calipers with ±0.01 mm of error.

During the experimental period, we chose three sunny days (each time from 10:00 to 14:00) to identify the range and relative frequency of different pollinator species, observing their behavior of pollination. The frequency of different pollinator species was recorded by observing bees presented in a defined area (2 × 2 m^2^) or along a transverse sector (50 m^2^) in the population. For the mechanical match between pollinators and flowers, we stochastically targeted a plant (or a flower) being probed (totally for 400 flowers), followed by recording its sex morph and observing whether the forager could touch its stigma. Three to five individuals of each pollinator species were collected for species identification and for morphological measurements including body length, thorax thickness (i.e. body thickness in the part of thorax), thorax width and tongue length (naturally extended length).

### Data analysis

We employed linear model in R (3.5.0) to determine difference in floral traits between sexual morphs in the gynodioecious *S. pratensis*. We calculated fruit set as fruit number per flower and seed set as seed (nutlet) number per flower per inflorescence. Generalized linear regression (GLM) was used to test the differences in fruit set and seed set (quasibinominal family, logit link function) between both morphs. We measured pollination efficiency of pollinators by calculating relative frequency of touching-stigma visits in total visits, and determined its difference between morphs with Pearson’s chi-squared test.

Multivariate regression analysis was used to estimate the strength and pattern of selection on the floral traits [55–57]. We employed the most complete regression model for estimates of different selection gradients (see [58]). The complete model includes linear and quadratic terms of each trait, and the products of pairs of all traits included. The partial regression coefficient of each trait serves as the main indicator of directional selection (β), and the double value of coefficient for a trait squared as a measure of nonlinear selection on the trait (γ, i.e. nonlinear selection gradient); and a significant coefficient of the product of a pair of traits as a measure of selection on combination of the two traits (i.e. correlational selection gradient) [58, 59]. We performed separate analyses for each sexual morph. Before the analyses, the seed number of each individual was standardized to the relative seed number (i.e. individual seed number / morph mean) as estimate of the female fitness component. Each trait value of an individual plant was the mean of 3 or 4 flowers. The trait values for each morph were standardized (i.e. mean = 0 and standard deviation = 1) by subtracting morph mean from the individual trait value and then divided by the standard deviation. Because of significant correlations of corolla length with tube length and corolla platform (each correlation coefficient > 0.86), we used relative platform (r-platform) rather than platform by dividing platform by corolla length in selection analysis, as a measurement of flower mouth opening (i.e. 1- tube/corolla). That is, we introduced flower number, style height, style exsertion, corolla and r-platform without corolla tube in the model for selection analysis. In addition, considering the effect of resource availability on plant’s size and thus on both the size of floral traits and fecundity, we introduced stalk diameter as a variable of plant size in the model to eliminate maternal effect on selection for traits [53, 60].

After establishing the full model, we further obtained the least adequate model by step regression. The significant terms in the full model were almost consistent in the number and sign with those in the least adequate model except that the significance was enhanced. Therefore, to maintain completeness and consistence of the models for two morphs, we presented the results from the full model in the study. Finally, we performed comparison of selection on floral traits between two sexual morphs, by establishing ANCOVA models with sexual morph as a factor. For all the models, we checked the normality of error distribution by Shapiro Test (W = 0.96, p = 0.19 for female group; W = 0.98, p = 0.55 for hermaphrodite group), the variance constancy by Non-constant Variance Score Test (Chisquare = 0.01, p = 0.92 for female group; Chisquare = 0.13, p = 0.71 for hermaphrodite group). We also assessed the assumptions of linear model using the Global Test with the Package gvlma in R, and each of assumptions was acceptable (p > 0.05). Kappa values for correlation matrix of the traits included in the models were 8.50 for female group, and 11.3 for hermaphrodite group, so there was no evidence for a problem of multi-collinearity. Finally, we illustrated selection gradients that were significantly different between two sexual morphs with the Added-Variable Plots [61]. Except for the standardization of variables, no transformations were done. For all data analysis, R version 3.5.0 [62] was used.

## Data Availability

Data generated or analyzed during this study are either included in this published article or are available from the corresponding author on reasonable request.

## Abbreviations

ANCOVA: Analysis of covariance
ANOVA: Analysis of variance
cl: corolla length
GLM: Generalized linear regression
pf: platform size
rua: reduced upper anther
se: style exsertion
sth: style height, i.e. the distance between the lower lip and the stigma
tl: corolla tube length
ufa: upper fertile anther
ula: upper lever arm
